# Supplementary material for: Deficiency of the Heterogeneous Nuclear Ribonucleoprotein U locus leads to delayed hindbrain neurogenesis
Source: Biol Open. 2023 Oct 10;12(10):bio060113. doi: 10.1242/bio.060113 (PMC10581386; doi:10.1242/bio.060113)
Supplement: Supplementary information [file biolopen-12-060113-s1.pdf]

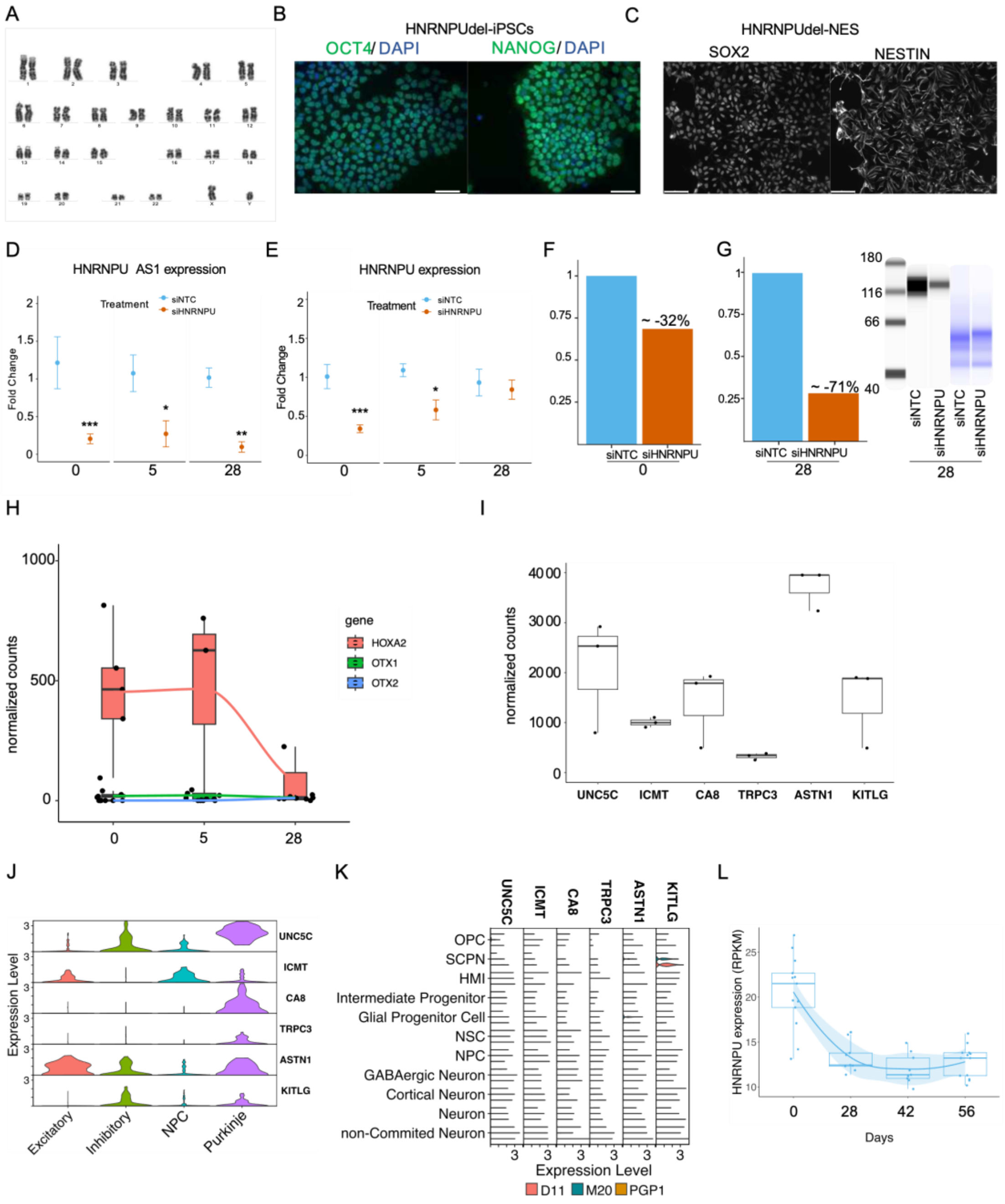

Fig. S1. Characterization of the two *HNRNPU* knockdown conditions. A) Karyotype of iPSCs obtained from the fibroblasts of the individual carrying the heterozygous deletion of HNRNPU, and from which the HNRNPU$_{del/+}$ cells were derived. **B)** Staining the HNRNPU$_{del/+}$ -iPSC cells with the pluripotency markers OCT4 and NANOG. Nuclei are counterstained with DAPI. Scale bar= 50µm. **C)** Immunostaining of SOX2 and NESTIN in HNRNPU$_{del/+}$ cells at D0. Scale bar= 50µm. **D-E)** *HNRNPU-AS1* (**D**) and *HNRNPU* (**E**) RNA expression after treatment with siHNRNPU at D0, D5 and D28. Bar plot normalized for each siNTC sample.

F-G) HNRNPU protein expression after treatment with siHNRNPU at D0 (**F**) and D28 (**G**). Bar plot normalized for each siNTC sample. Capillary western blot representation (right) of the samples at D28 probed with antibody against HNRNPU and total protein quantification in the following lanes. **H)** Expression of embryonic markers *HOXA2*, *OTX1*, *OTX2* in CTRL cells at D0, D5 and D28. **I)** Expression of cerebellar markers (*UNC5C*, *ICMT*, *CA8*, *TRPC3*, *ASTN1*, *KITLG)* in CTRL cell line at D28. **J)** Expression of cerebellar markers among subtypes of neuronal cells in our previously published scRNA-seq data(Becker et al., 2020) at D28 visualized in violin plot. NPC=Neural progenitor cell. **K)** Expression of cerebellar markers among cell types and across samples in organoid scRNA-seq data(Ressler et al., 2023). D11=*HNRNPU* deficient organoids with a heterozygous frameshift mutation, M20=*HNRNPU* deficient organoids with a heterozygous premature termination codon, PGP1=Control organoids. **L)** *HNRNPU* RNA expression in neurotypical samples from published iPSC-neuronal study dataset(Burke et al., 2020). The day 0 refers to rosette forming cells comparable to NES cells and the following time points are days in differentiation.

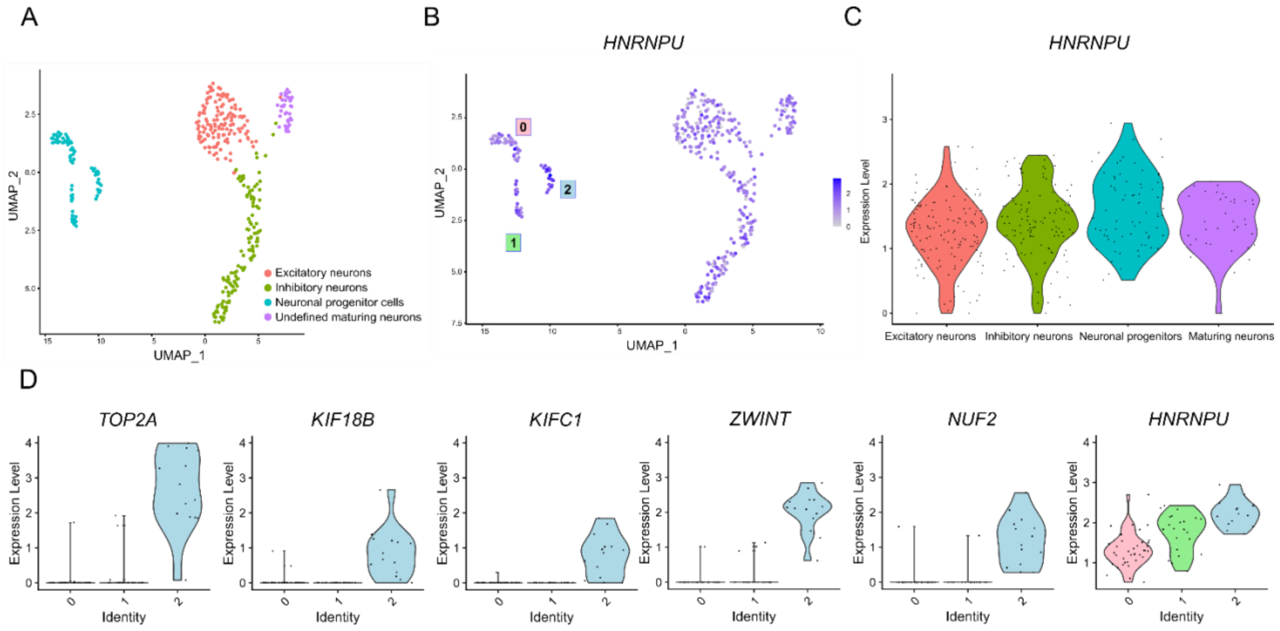

Fig. S2. *HNRNPU* expression is higher in proliferating progenitor cells. A) UMAP dimensional reduction of scRNA-seq data from previously published cells at D28, obtained with the same differentiation protocol as the cell model in study(Becker et al., 2020). The different cell type populations are highlighted. **B)** Feature plot of HNRNPU expression in the cell populations from the scRNA-seq analysis **C)** Violin plot of HNRNPU expression in each cell type from the scRNA-seq analysis. **D)** Expression of proliferation markers in the subclusters defining the NPCs subpopulation from the scRNA-seq analysis. 0-1-2 refer to the NPCs subpopulations showed in H. In the figure $HNRNPU_{del/+}$ samples are indicated as "HNRNPUdel".

Biology Open • Supplementary information

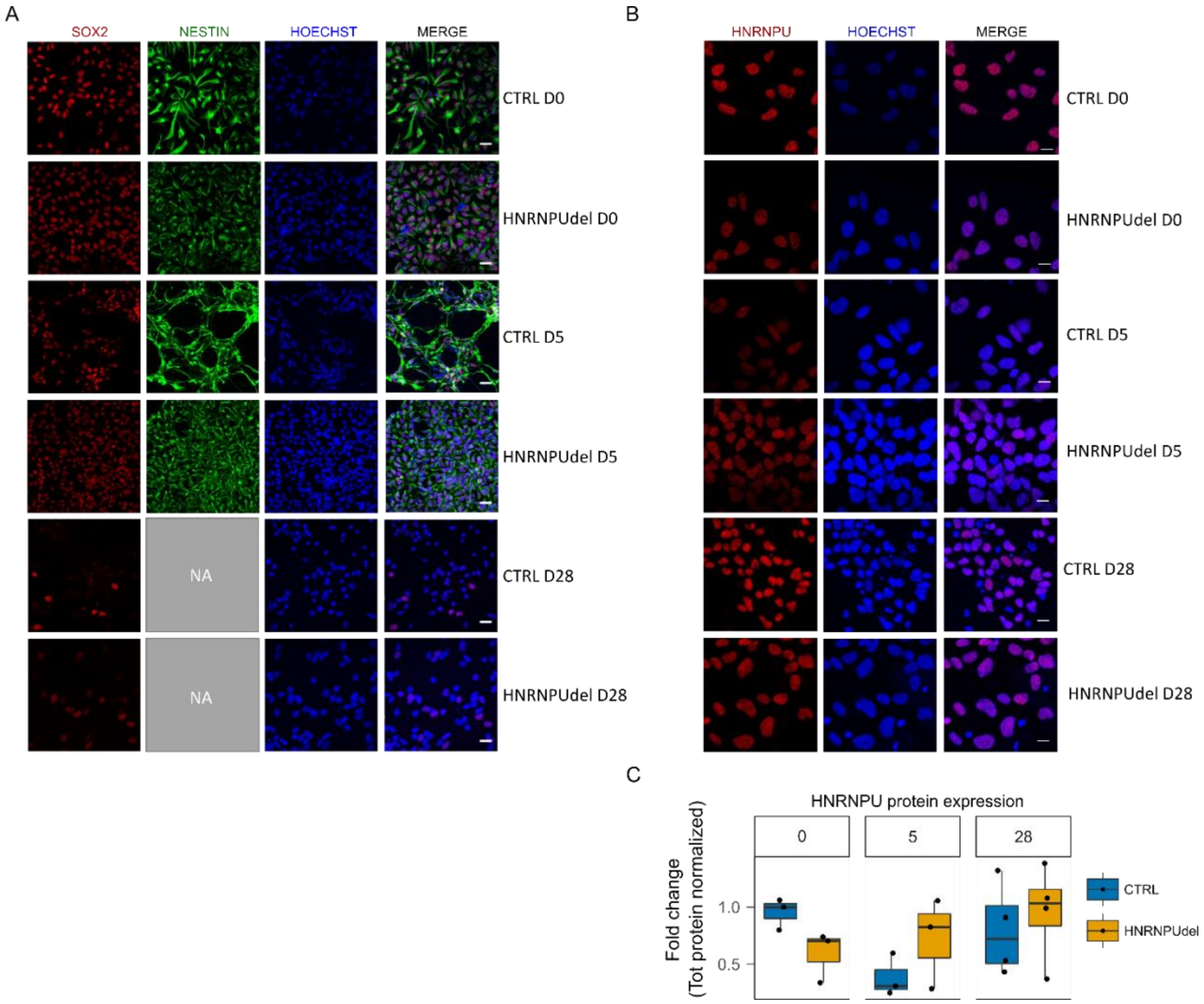

Fig. S3. HNRNPU protein localization and expression during neuronal differentiation. **A)** Immunostaining of HNRNPU in red, Hoechst in blue and merge at D0, D5 and D28 in CTRL and $HNRNPU_{del/+}$. Scale bar= 10 µm. **B)** HNRNPU protein quantification from capillary western blot during differentiation in CTRL and $HNRNPU_{del/+}$ (n=3-5). In the figure $HNRNPU_{del/+}$ samples are indicated as "HNRNPUdel".

Biology Open • Supplementary information

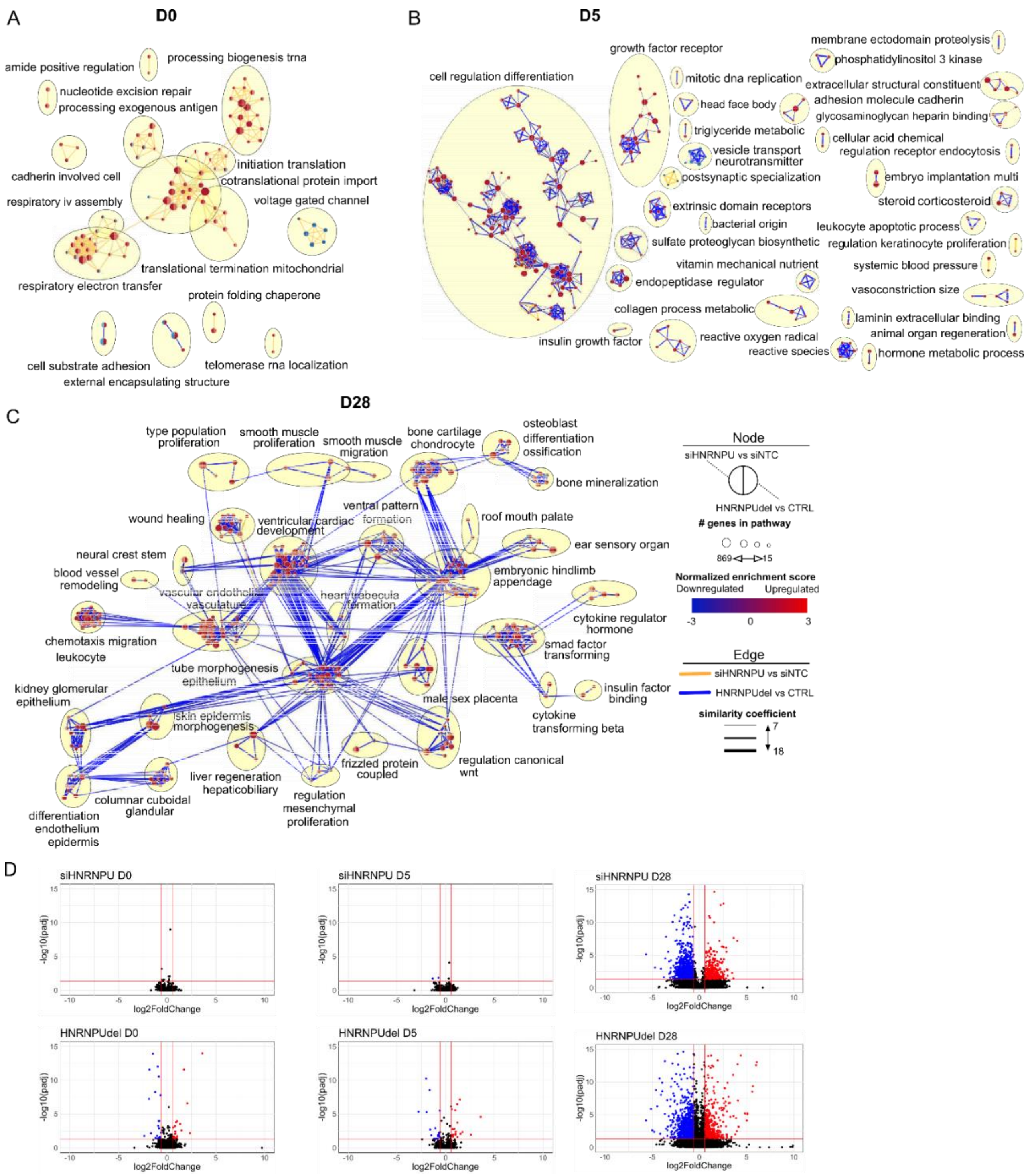

Fig. S4. Extended differential pathway enrichment under HNRNPU downregulation during neurodevelopment. A-B) All the upregulated and downregulated pathways, respectively, at D0 and D5. **C)** Upregulated pathways at D28 related to general development. For each node, the left half indicates the siHNRNPU versus siNTC enrichment and the right half the $HNRNPU_{del/+}$ versus CTRL enrichment. The color of the edge indicates which of the datasets significantly contributed to the pathway call. **D)** Volcano plots of DEU exon bins at D0, D5 and D28 for both HNRNPU deficient conditions. The plots are zoomed in, and some highly significant exon bins are thus excluded from the D28 plots to enhance readability. In red is the upregulated DEU, and in blue is the downregulated DEU. The vertical lines indicate the threshold for significance (p adj < 0.05 and |log2FoldChange|>0.58). In the figure HNRNPUdel/+ samples are indicated as “HNRNPUdel”.

Biology Open • Supplementary information

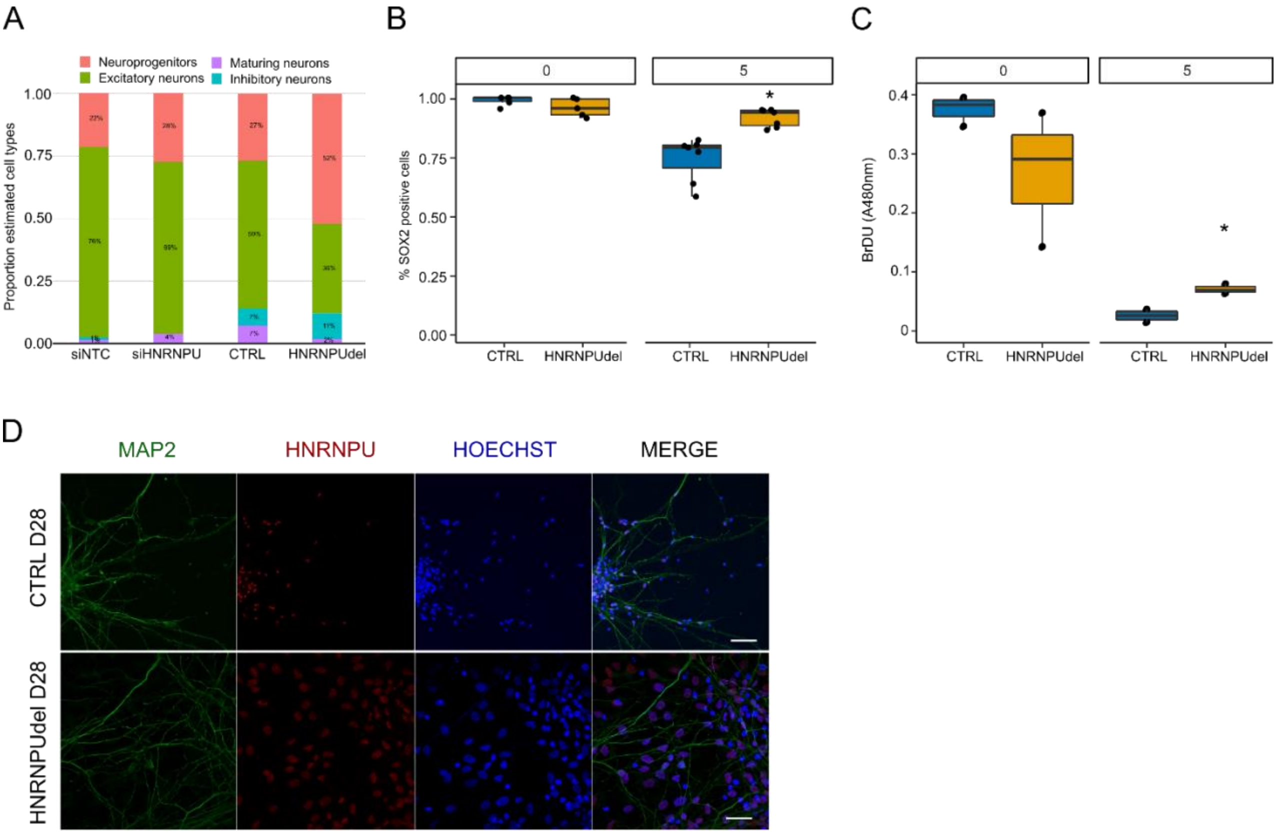

Fig. S5. Delayed maturation and higher progenitor phenotype under HNRNPU downregulation during the differentiation time course

A) Estimated proportion of the different cell types at D28 after deconvolution analysis. **B)** SOX2 positive cells at D0 and D5 in CTRL and $HNRNPU_{del/+}$. **C)** BrdU signal at D0 and D5 in CTRL and $HNRNPU_{del/+}$. **D)** MAP2, HNRNPU and Hoechst staining of CTRL and $HNRNPU_{del/+}$ at D28. Scale bar= 50µm. *p<0.05. In the figure $HNRNPU_{del/+}$ samples are indicated as “HNRNPUdel”.

Biology Open • Supplementary information

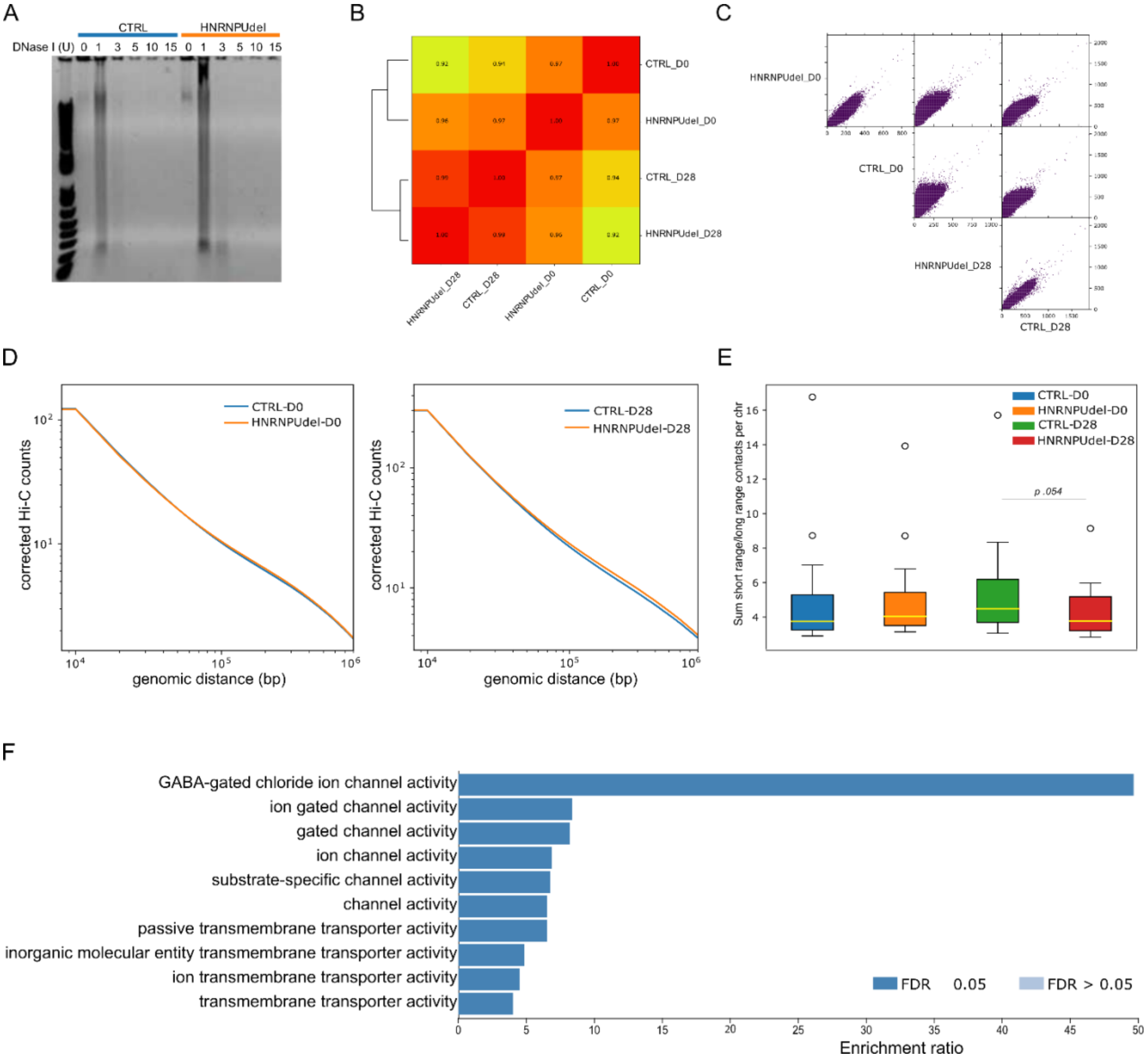

Fig. S6. Extended effects of HNRNPU downregulation on chromatin organization. A) DNaseI sensitivity assay in CTRL and HNRNPU$_{del/+}$. **B)** Heatmap of the correlation matrixes of the HiC samples. **C)** Pearson's correlation matrixes of the HiC samples. **D)** Corrected HiC counts at a specific genomic distance at D0 (left) and D28 (right). **E)** Boxplot of short range/long range HiC contact per chromosome. **F)** Pathways enriched in the "concordant A to B" genes, according to Over-representation analysis from WebGestalt. In the figure HNRNPU$_{del/+}$ samples are indicated as "HNRNPUdel".

Biology Open • Supplementary information

Table S1. Phenotype information of monozygotic twin pair carrying heterozygous deletion affecting HNRNPU locus

	Twin 1	Twin 2
Birth week and delivery mode	36 + 4 with caesarean section	
Birth measures	Weight: 2310 g, height: 44 cm<br>Head circumference: 34<br>Apgar score: 9, 9, 10	Weight: 2340g, height: 44 cm<br>Head circumference: 33<br>Apgar score: 8, 10, 10
Problems in infancy	Feeding problems<br>Jaundice<br>Frequent ear infections<br>Eczema<br>Scarlet fever<br>Phototherapy after birth	Feeding problems<br>Jaundice<br>Frequent ear infections<br>Eczema<br>Scarlet fever<br>Oxygen therapy after birth
Early development	Language: Delayed (first words 84 months old, sentences 116 months)<br>Motoric: Delayed (walked at 4 year of age)<br>Food: Problems with breast-feeding and with solid food<br>Toilet training delayed (not obtained by 9 years of age)<br>First noted abnormalities: 6 months	Language: Delayed (first words 54 months old, sentences 72 months)<br>Motoric: Delayed (walked at 3 year of age)<br>Food: Problems with breast-feeding and with solid food<br>Toilet training completed by 5 years of age<br>First noted abnormalities: 6 months
Somatic diagnoses in infancy/childhood	Heart problem (ventricular septal defect, operated)<br>Inguinal hernia (operated)<br>Fever-induced seizures<br>Lyme disease<br>Carrier of Methicillin-resistant Staphylococcus aureus (MRSA)	Irritable bowel disorder<br>Inguinal hernia (operated)<br>Fever-induced seizures<br>Carrier of Methicillin-resistant Staphylococcus aureus (MRSA)
Diagnosis in research study RATSS	299.00 Autism spectrum disorder<br>319 Intellectual disability mild<br>307.20 Unspecified tic disorder<br>307.6 Enuresis<br>307.7 Encopresis	299.00 Autism spectrum disorder<br>319 Intellectual disability mild<br>780.52 Insomnia disorder
Medications at time for assessment	Levotyroxin<br>Movicol<br>Alimemazine	Levotyroxin
IQ screen (LEITER-R)	IQ 60	IQ 58
Adaptive functioning (ABAS 2)	GAF 40	GAF 40
Autism symptoms	ADOS-2: total score 23, severity score 9 (classified as ASD)<br>ADI-R: above cut-off (classified as ASD)<br>SRS total score: 141	ADOS-2: total score 26, severity score 10 (classified as ASD)<br>ADI-R: above cut-off (classified as ASD)<br>SRS total score: 140
ADHD symptoms	Connors 3 (raw score)	Connors 3 (raw score)

	Global Index: 11<br>ADHD/Inattentive: 14<br>ADHD/Hyperactive-Impulsive: 7	Global Index: 15<br>ADHD/Inattentive: 10<br>ADHD/Hyperactive-Impulsive: 7
General comments by clinical geneticists	Abnormal eye contact<br>Monotone speech<br>Repetitive movements	Abnormal eye contact<br>Monotone speech<br>Repetitive movements<br>Echolalia
Morphological findings	Macrocephaly (head circumference 54 cm)<br>Wide space between teeth and late second teeth in upper jaw<br>Flat feet<br>Tapered fingers<br>Bite marks left hand	Macrocephaly (head circumference 53.8 cm)<br>Wide space between teeth and late second teeth in upper jaw<br>Flat feet<br>Tapered fingers<br>Bite marks left hand<br>Overweight
Parental education	Mother: University; Father: Elementary school	

ABAS, Adaptive behaviour assessment system; ADHD, Attention-deficit/hyperactivity disorder; ADI-R, Autism Diagnostic Inventory-Revised; ADOS, Autism Diagnostic Observation Schedule—Second Edition; ASD, Autism Spectrum Disorder; GAF, Global Assessment of Functioning; IQ, Intelligence quotient; SRS, Social Responsiveness Scale

Table S2. DeSeq2 results after RNAseq Bulk sequencing. A) comparison at D0, siHNRNPU and siNTC; B) comparison at D5, siHNRNPU and siNTC; C) comparison at D0, HNRNPUdel/+ and CTRL; D) comparison at D5, HNRNPUdel/+ and CTRL; E) comparison at D28, siHNRNPU and siNTC; F) comparison at D28, HNRNPUdel/+ and CTRL.

Click here to download Table S2

Table S3. GSEA analysis of pre ranked genes from the DeSeq2 analysis of siHNRNPU compared to siNTC and HNRNPUdel/+ compared to CTRL at each time point. GSEA positively enriched pathways represent the pathways upregulated in the HNRNPU deficient conditions, and the negatively enriched are the downregulated. A-B) Upregulated and downregulated pathways at D0 in the comparison of siHNRNPU versus siNTC, respectively. C-D) Upregulated and downregulated pathways at D0 in the comparison of HNRNPUdel/+ versus CTRL, respectively. E-F) Upregulated and downregulated pathways at D5 in the comparison of siHNRNPU versus siNTC, respectively. G-H) Upregulated and downregulated pathways at D5 in the comparison of HNRNPUdel/+ versus CTRL, respectively. I-J) Upregulated and downregulated pathways at D28 in the comparison of siHNRNPU versus siNTC, respectively. K-L) Upregulated and downregulated pathways at D28 in the comparison of HNRNPUdel/+ versus CTRL, respectively.

Click here to download Table S3

Table S4. Enrichment in NDD gene lists of genes affected by HNRNPU deficiency at different levels of regulation (DEG, DEU, compartment organization) and different time points, by hypergeometric test; the signiicant enrichments are highlighted (FDR<0.05) m= number of genes in the NDD gene list; q= number of genes in common between the NDD gene list and the gene list under analysis -1. ASD=Autism spectrum disorder ID= Intellectual disability; EPI= Epilepsy; DD= developmental disorder

Click here to download Table S4

Table S5. DEXSeq results, for differential exon usage analysis. Significantly differentially used exon bins are displayed. A)comparison at D5, siHNRNPU and siNTC; B)comparison at D28, siHNRNPU and siNTC; C) comparison at D0, HNRNPUdel/+ and CTRL; D) comparison at D5, HNRNPUdel/+ and CTRL; E) comparison at D28, HNRNPUdel/+ and CTRL.

Click here to download Table S5

Table S6. Gene set enrichment of DEU genes at D28 in the comparisons siHNRNPU versus siNTC (A) and HNRNPUdel/+ versus CTRL (B).

Click here to download Table S6

Table S7. Concordant genes in the compartment regions that switch from A to B or B to A in HNRNPUdel/+ versus CTRL at D28.

Click here to download Table S7
